# Nerve conduction study of the association between glycemic variability and diabetes neuropathy

**DOI:** 10.1186/s13098-018-0371-0

**Published:** 2018-09-12

**Authors:** Miho Akaza, Itaru Akaza, Tadashi Kanouchi, Tetsuo Sasano, Yuki Sumi, Takanori Yokota

**Affiliations:** 1 Respiratory and Nervous System Science, Biomedical Laboratory Science, Graduate School of Medical and Dental Sciences, Tokyo Medical and Dental University, 1-5-45 Yushima, Bunkyo-ku, Tokyo, 113-8519 Japan; 2 Internal Medicine of Endocrinology and Metabolism, Shuuwa General Hospital, 1200 Yaharashinden, Kasukabe-shi, Saitama, Japan; 3 Clinical Laboratory, Tokyo Medical and Dental University Medical Hospital, 1-5-45 Yushima, Bunkyo-ku, Tokyo, 113-8519 Japan; 4 Cardiovascular Physiology, Biomedical Laboratory Science, Graduate School of Medical and Dental Sciences, Tokyo Medical and Dental University, 1-5-45 Yushima, Bunkyo-ku, Tokyo, 113-8519 Japan; 5 Department of Neurology and Neurological Science, Tokyo Medical and Dental University Graduate School, 1-5-45 Yushima, Bunkyo-ku, Tokyo, 113-8519 Japan

**Keywords:** Glycemic variability, Continuous glucose monitoring, Diabetes peripheral neuropathy, Nerve conduction study

## Abstract

**Background:**

It remains unclear whether glycemic variability is related to diabetes microvascular disease, especially diabetes peripheral neuropathy (DPN). We investigated the association between glycemic variability and DPN with type 1 or 2 diabetes.

**Methods:**

Forty patients (23 males and 17 females; aged 34–79 years) underwent continuous glucose monitoring (CGM) and a nerve conduction study (NCS). Glycemic variability was estimated by mean amplitude of glycemic excursions (MAGE) in CGM. DPN was quantitatively evaluated by NCS in the median, tibial, sural and medial plantar nerves.

**Results:**

MAGE had a significantly positive correlation with disease duration and low-density lipoprotein cholesterol level (r = 0.462, p = 0.003; and r = 0.40, p = 0.011, respectively), and a significantly negative correlation with BMI and medial plantar compound nerve action potential amplitude (r = − 0.39, p = 0.012; and r = − 0.32, p = 0.042, respectively). Multivariate linear regression analysis with adjustment for clinical background showed that MAGE (β = − 0.49, p= 0.007) was independently associated with a higher risk of medial plantar neuropathy.

**Conclusions:**

Glycemic variability may be an independent risk factor for DPN.

## Background

Current treatment strategies for diabetes aim to control glucose levels to prevent the development of diabetes-related complications. Hemoglobin A1c (HbA1c) testing represents the gold standard for the assessment of long-term glycemic control. An acceptable HbA1c level, as defined by the American Diabetes Association (ADA), is 7% or lower [1]. However, intensive glucose control led to increased mortality compared with standard therapy in the ACCORD study [2], leading to a review of the strategies for glycemic control. Glycemic disorders in diabetes are not limited to fasting and postprandial hyperglycemia, but can be extended to glycemic variability in an upward (postprandial glucose increments) and downward (interprandial glucose decrements) direction [3]. Continuous glucose monitoring (CGM) allows glycemic variability to be evaluated more exactly compared with conventional self-monitoring blood glucose (SMBG). Glycemic variability has been suggested as an independent risk factor for diabetes complications [4–6]. Diabetes peripheral neuropathy (DPN), one of the most common microvascular complications of diabetes, is associated with foot ulceration, amputation and a significant reduction in quality of life [7, 8]. A correlation between DPN and glycemic variability evaluated by CGM has previously been reported [9, 10], with DPN diagnosed on the basis of patient symptoms and neurological examination. However, DPN develops before symptoms such as numbness and pain appear. A nerve conduction study (NCS) can quantitatively detect peripheral nerve dysfunction, even in the pre-symptomatic stage of DPN onset. We therefore evaluated peripheral nerve function in diabetes patients using NCS to investigate the association between glycemic variability assessed by CGM and DPN assessed by NCS.

## Subjects

Forty outpatients at Shuuwa General Hospital with type 1 or type 2 diabetes (23 males and 17 females, aged 34–79 years) were enrolled in this study between October 2015 and March 2017. The diagnosis of diabetes was based on the criteria of the Japan Diabetes Society [11]. Patients aged over 80 years were excluded because the NCS normal reference ranges in our institution were based on individuals aged less than 80 years. Patients with severe renal impairment (estimated glomerular filtration rate [eGFR] < 15 mL/min/1.73 m^2^ or undergoing renal replacement therapy), severe hepatic function and other peripheral neuropathies were also excluded. Thirteen patients with type 1 diabetes (T1D) had a history of positive antibodies, including GAD-ab and/or IA2-ab and severely impaired insulin secretion (serum C-peptide immunoreactivity < 0.6 ng/mL and/or 24-h urine collection C-peptide immunoreactivity < 20 μg/day), and were treated with a basal-bolus injection of insulin. Twenty-seven patients with type 2 diabetes (T2D) were treated with oral hypoglycemic agents, glucagon-like peptide-1 receptor agonist, or insulin. All patients received education about diabetes and had stable glycemic control with the same medication for diabetes (as demonstrated by two HbA1c readings differing by no more than 1%) during the 6 months prior to the start of the study. Age, sex, height, body weight, BMI, blood pressure in the office setting, duration of diabetes, medications, and comorbidities were recorded at the beginning of the study. Duration of diabetes was defined as the known years of disease at the time of recruitment. Macrovascular disease was assessed from clinical history of myocardial infarction, angina pectoris, coronary artery surgery, angioplasty, and stroke. Microalbuminuria was defined as a urinary albumin excretion level between 30 and 299 mg/g creatinine on at least two of three occasions; macroalbuminuria was defined as a urinary albumin excretion level > 300 mg/g creatinine on at least two of three occasions. Retinal lesions were classified as mild, moderate, or severe nonproliferative diabetes retinopathy (DR), or proliferative DR, according to international clinical diabetes retinopathy and diabetes macular edema disease severity scales [12].

## Methods

### Evaluation of glycemic variability

A CGM sensor (IPro2^®^, Medtronic, Minimed, USA) was inserted into the subcutaneous abdomen and measured glucose concentration in the interstitial fluid every 5 min for 7 days. During CGM monitoring, blood glucose levels were determined a minimum of four times per day using an SMBG device (Medisafe Mini, Terumo, Japan) to convert the CGM data on glucose concentration in interstitial fluid to blood glucose data. A total of 288 blood glucose data points could therefore be obtained daily by CGM. Mean amplitude of glycemic excursion (MAGE), which can quantify major variations in glycemic control but exclude minor variations, was used to assess intra-day glycemic variability [13] and was calculated as the arithmetic mean of the difference between consecutive peaks and nadirs where the difference was > 1SD. Five-day average MAGE over days 2–6 was obtained. Based on the CGM data, the presence of hypoglycemia, defined as glucose level < 70 mg/dL for 15 min in 1 day, was also determined.

### Nerve conduction study

NCS was performed in the median, tibial, sural, and medial plantar nerves on the nondominant hand side using a Neuropack S1 (Nihon Kohden, Tokyo, Japan) with the bandpass filter set at 5 Hz–5 kHz. Compound muscle action potential (CMAP) was recorded from a pair of surface cup electrodes placed over the target muscle (abductor pollicis brevis for the median nerve and abductor hallucis for the tibial nerve) using the belly-tendon method. Square pulse supramaximal electrical stimuli with a duration of 0.5 ms were delivered at the wrist and elbow to the median nerve and at the ankle and popliteal fossa to the tibial nerve. Twenty consecutive F waves were also recorded in each nerve. Sensory nerve action potential (SNAP) was antidromically recorded from a pair of ring electrodes placed over the distal and proximal interphalangeal joints of the index finger for the median nerve, and from a pair of surface cup electrodes placed at the points posterior to the lateral malleolus and 3 cm distal to it for the sural nerve. Square pulse supramaximal electrical stimuli with a duration of 0.2 ms were delivered at the wrist and elbow for the median nerve and at the midcalf, 12 cm proximal to the recording electrode, for the sural nerve. In the medial plantar nerve, compound nerve action potential (CNAP) was orthodromically recorded from a pair of surface cup electrodes placed over the tibial nerve at the ankle, posterior to the medial malleolus. Stimulation was carried out on the sole, placing the anode just lateral to the first metatarsal head and the cathode 2.5 cm proximal to it. Skin temperature was maintained above 32 °C in the upper limbs and above 31 °C in the lower limbs.

Measurements included peak-to-peak amplitude of the response, distal latency, conduction velocity, and minimal F wave latency (FLmin). Given that FLmin correlates with subject height, we evaluated it as a z score, calculated with the following formula: z score = (measured FLmin − estimated FLmin by height)/half of a one-sided 95% confidence interval of estimated FLmin by height. The estimated FLmin and 95% confidence interval were based on linear regression analysis between FLmin and height in healthy subjects (data not shown).

### Statistical analysis

Statistical analysis was carried out using SPSS version 24.0 (IBM, Armonk, NY, USA), and the results were expressed as mean ± standard deviation (SD) or median with interquartile range as appropriate, according to data distribution. We used Student’s t-test, the Mann–Whitney U test, or the χ^2^ test for group comparisons. The Pearson correlation coefficient or Spearman’s rank correlation coefficient were used to evaluate correlations between variables, where appropriate. Multiple linear regression analyses were performed to determine the independent factors associated with NCS parameters, using the following covariates: MAGE, gender, age, type of diabetes, duration of diabetes, HbA1c, low-density lipoprotein cholesterol (LDL), systolic blood pressure (SBP), and BMI. All p values < 0.05 were considered statistically significant.

## Results

**Fig. 1:**
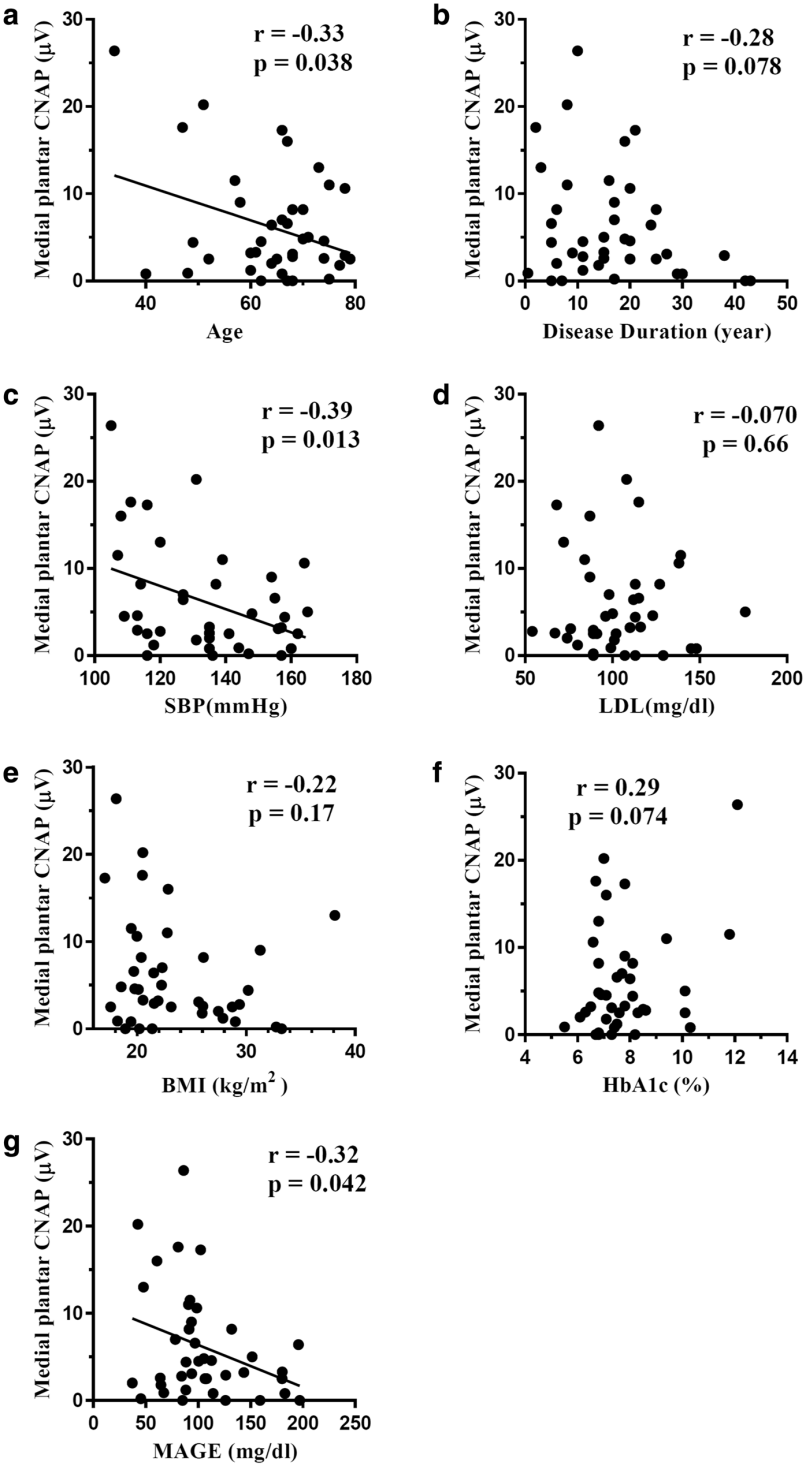
Univariate linear regression analysis of medial plantar CNAP amplitude and clinical parameters including age (**a**), disease duration (**b**), SBP (**c**), LDL (**d**), BMI (**e**), HbA1c (**f**), and MAGE (**g**). Medial plantar CNAP amplitude was shown to have a negative correlation with age, SBP, and MAGE

Table 1 shows the baseline clinical characteristics, laboratory data, and NCS results of the 40 patients who underwent CGM. Patients with T2D had higher BMI and lower LDL than those with T1D (p = 0.03 and p = 0.005). There was no difference in other factors including MAGE and NCS results between T1D and T2D patients. Six out of 40 patients had macrovascular disease, but no relationship was observed between the existence of macrovascular disease and HbA1c or MAGE. Eight of 40 patients with severe nonproliferative DR or proliferative DR had a tendency toward higher MAGE, but this association was not significant (99.5 ± 42.3 vs 127.5 ± 33.8, p = 0.091). Seventeen of 40 patients with hypoglycemia had significantly higher MAGE (139.3 ± 39.1 vs 86.6 ± 30.6, p < 0.001) and a tendency toward lower HbA1c, which was not significant (7.4 ± 1.0 vs 8.4 ± 1.8, p = 0.068). Table 2 shows the univariate linear regression analysis for the relationship between glycemic variability and clinical characteristics, laboratory data, and NCS results. MAGE had a significantly positive correlation with disease duration and LDL (r = 0.462, p = 0.003; and r = 0.40, p = 0.011, respectively). MAGE had a tendency toward a positive correlation with HbA1c, but this effect was not significant (r = 0.292, p = 0.067). MAGE had a significantly negative correlation with BMI and medial plantar CNAP amplitude (r = − 0.39, p = 0.012; and r = − 0.32, p = 0.042, respectively). As shown in Fig. 1, medial plantar CNAP amplitude had a negative correlation with age (r = − 0.33, p = 0.038), SBP (r = − 0.39, p = 0.013), and MAGE (r = − 0.32, p = 0.042).

**Table 1:** Comparison of clinical characteristics, laboratory data, and NCS results between patients with type 1 and type 2 diabetes

	All subjects	Type 1 diabetes	Type 2 diabetes	p value
n	40	13	27	
Age (year)	64.1 ± 10.4	58.9 ± 14.4	66.7 ± 6.8	0.087
Disease duration (year)	16.4 ± 10.4	17.7 ± 12.6	15.7 ± 9.4	0.58
Number of patients with hypoglycemia	17	7	10	0.31
HbA1c (%)	7.8 ± 1.4	8.1 ± 2.0	7.6 ± 1.0	0.37
BMI (kg/m^2^)	23.5 ± 5.0	21.1 ± 3.92	24.7 ± 5.1	0.03
SBP (mmHg)	134 ± 18	131.9 ± 20.8	134.9 ± 17.4	0.64
TG (mg/dL)	146 ± 105	117.2 ± 95.1	160.1 ± 108.5	0.23
LDL (mg/dL)	103 ± 25	118.6 ± 19.9	95.9 ± 24.0	0.005
eGFR (mL/min/1.73 m^2^)	67.9 ± 19.9	75.0 ± 22.9	64.5 ± 17.7	0.12
UAE (mg/gCre)	10.5 [6.5–28.6]	9.6 [6.7–23.0]	11.8 [5.4–37.3]	0.65
MAGE (mg/dL)	105 ± 41.9	119.6 ± 44.4	98.0 ± 39.5	0.13
Median CMAP amp (mV)	14.4 ± 3.4	15.0 ± 4.2	14.2 ± 3.0	0.49
Median DL (m/s)	3.93 ± 0.82	3.80 ± 0.61	3.99 ± 0.92	0.50
Median MCV (m/s)	53.5 ± 3.72	53.0 ± 3.8	53.7 ± 3.7	0.56
Median F z score	2.19 ± 1.49	1.77 ± 1.54	2.39 ± 1.45	0.23
Median wrist SNAP amp (µV)	29.7 ± 13.9	33.0 ± 17.6	28.2 ± 11.8	0.31
Median SCV (m/s)	47.0 ± 10.7	48.5 ± 7.1	46.3 ± 12.1	0.54
Tibial CMAP amp (mV)	18.8 ± 7.1	20.6 ± 6.8	17.9 ± 7.3	0.26
Tibial MCV (m/s)	42.5 ± 3.71	43.5 ± 3.8	42.0 ± 3.6	0.21
Tibial F z score	2.53 ± 1.72	2.10 ± 1.32	2.73 ± 1.87	0.28
Sural SNAP amp (µV)	14.0 ± 8.6	17.0 ± 11.0	12.6 ± 7.3	0.20
Sural SCV (m/s)	46.6 ± 4.7	44.9 ± 5.3	47.4 ± 4.2	0.11
Medial plantar CNAP amp (µV)	6.14 ± 6.28	7.28 ± 7.74	5.58 ± 5.53	0.43
Medial plantar NCV (m/s)	48.4 ± 6.2	46.8 ± 5.4	49.2 ± 6.5	0.26

Data: mean ± SD or median [interquartile range], unpaired *t*-test or Mann–Whitney U test

Hypoglycemia: glucose level > 70 mg/dL for 15 min or longer

*HbA1c* glycated hemoglobin A1c, *BMI* body mass index, *SBP* systolic blood pressure, *TG* triglyceride, *LDL* low-density lipoprotein cholesterol, *eGFR* estimated glomerular filtration rate, *UAE* urinary albumin excretion, *MAGE* mean amplitude of glycemic excursions, *CMAP* compound muscle action potential, *SNAP* sensory nerve action potential, *CNAP* compound nerve action potential, amp: amplitude, *DL* distal latency, *MCV* motor nerve conduction velocity, *SCV* sensory nerve conduction velocity, *NCV* nerve conduction velocity

**Table 2:** Relationship between MAGE and clinical variables by univariate regression analysis

	r	Spearman ρ	p value
Age (years)	0.028		0.86
Disease duration (years)	0.462		0.003
HbA1c (%)	0.292		0.067
BMI (kg/m^2^)	− 0.39		0.012
SBP (mmHg)	0.045		0.78
TG (mg/dL)	− 0.18		0.27
LDL (mg/dL)	0.40		0.011
eGFR (mL/min/1.73 m^2^)	0.014		0.70
UAE (mg/gCre)		0.13	0.41
Median CMAP amp (mV)	− 0.098		0.55
Median DL (m/s)	0.032		0.84
Median MCV (m/s)	− 0.27		0.088
Median F latency z score	0.057		0.72
Median wrist SNAP (µV)	− 0.228		0.158
Median SCV (m/s)	− 0.00		0.99
Tibial CMAP amp (mV)	0.12		0.46
Tibial MCV (m/s)	− 0.043		0.79
Tibial F z score	− 0.25		0.12
Sural SNAP (µV)	− 0.15		0.37
Sural SCV (m/s)	0.055		0.74
Medial plantar CNAP amp (µV)	− 0.32		0.042
Medial plantar NCV (m/s)	− 0.033		0.849

*HbA1c* glycated hemoglobin A1c, *BMI* body mass index, *SBP* systolic blood pressure, *TG* triglyceride, *LDL* low-density lipoprotein cholesterol, *eGFR* estimated glomerular filtration rate, *UAE* urinary albumin excretion, *MAGE* mean amplitude of glycemic excursions, *CMAP* compound muscle action potential, *SNAP* sensory nerve action potential, *CNAP* compound nerve action potential, amp: amplitude, *DL* distal latency, *MCV* motor nerve conduction velocity, *SCV* sensory nerve conduction velocity, *NCV* nerve conduction velocity

Finally, we performed multiple linear regression analyses to confirm the association of NCS parameters with MAGE, with adjustment for clinical background. There was a significant regression (R^2^ = 0.49, *p* = 0.008) based on medial planter CNAP amplitude as a dependent variable and gender, type of diabetes, age, duration of diabetes, SBP, LDL, HbA1c, and MAGE as independent variables. MAGE (β = − 0.49, *p* = 0.007) was independently associated with a higher risk of medial plantar neuropathy in our patient population (Table 3). There was no significant regression for glycemic variability using other NCS parameters as dependent variables.

**Table 3:** Multivariate linear regression analysis for independent factors associated with medial plantar CNAP amplitude

Covariates	Standardized β	p values
Gender	− 0.060	0.72
Type of diabetes	− 0.011	0.95
Age	− 0.23	0.14
Duration of diabetes	− 0.061	0.70
BMI	− 0.34	0.037
SBP	− 0.23	0.18
LDL	0.020	0.92
HbA1c	0.28	0.074
MAGE	− 0.49	0.007

R^2^ = 0.49, p = 0.008

## Discussion

This is the first study to show a quantitative correlation between glycemic variability assessed by CGM and axonal loss of the medial plantar nerve evaluated by NCS. Multivariate linear regression analysis of our data suggests that the glycemic variability could be an independent risk factor for DPN. Previous studies have shown that not only fasting plasma glucose or HbA1c level on office visit, but also variability within these parameters was related to the risk of microvascular complications in diabetes [14–18]. However, the variations in fasting plasma glucose or HbA1c do not necessarily correspond to the variations in all time plasma glucose. Indeed, in the present study, patients with stable HbA1c showed variations in MAGE, the index of glycemic variability calculated using CGM data. CGM appears to be superior to the monitoring of fasting plasma glucose or HbA1c for the evaluation of glycemic variability. An association between microvascular complications and glycemic variability assessed by CGM has previously been reported [9, 10, 19–21]. Xu et al. showed that glycemic variability assessed by CGM was associated with DPN in patients with type 2 diabetes with well-controlled HbA1c, although the diagnosis of DPN was based only on clinical symptoms and signs. DPN develops prior to symptoms such as numbness and pain. However, NCS, as used in the present study, can detect peripheral nerve dysfunction even in early-stage disease and allow neuropathy to be quantitatively evaluated.

Sural nerve involvement in DPN is well established; however, glycemic variability evaluated by MAGE did not correlate with sural SNAP amplitude but did correlate with medial plantar CNAP amplitude in the present study. This may relate to the fact that some of our patients were in an earlier stage of DPN. The most common type of DPN is distal symmetrical polyneuropathy, and the examined segment of sural nerve is located more proximal than that of the medial plantar nerve. Therefore, NCS abnormalities in the sural nerve are detected at a later stage of DPN than those in the medial plantar nerve. Assessment of the medial plantar nerve in addition with conventional NCS increases the sensitivity of the detection of neuropathy, leading to earlier diagnosis [22–24]. In the present study, only 13 of 40 patients (32.5%) had a sural nerve abnormality (normal range > 10.3 μV), while 27 patients (67.5%) had a medial plantar nerve abnormality (normal range > 7.8 μV). This finding indicates that more than half of patients with DPN were in an earlier stage of the disease.

The potential mechanisms of DPN are reportedly associated with a number of risk factors, including the degree of hyperglycemia, lipid disorders, high blood pressure, and diabetes duration [25–27]. Our study suggests that glycemic variability is also an independent risk factor for DPN. Although both the medial plantar CNAP amplitude and MAGE correlated with disease duration by univariate linear regression analysis, only MAGE appeared to be an independent risk factor for the involvement of the medial plantar nerve by multivariate analysis. Our results show that CNAP amplitude of the medial plantar nerve decreases with age, which is well established, although no correlation between age and MAGE was observed. Multivariate analysis revealed that MAGE was independent of age as a risk factor for involvement of the medial plantar nerve.

It has been pointed out that MAGE has potential for errors in the calculation process [13]. Several indicators have been proposed for glycemic variability although the optimal indices remain controversial [28]. In the present study, we evaluated glycemic variability in outpatients with CGM under uncontrolled and free behavior. We selected MAGE as an indicator of glycemic variability as we considered it best reflects the original glucose spike, by excluding the effect of small blood glucose fluctuations even in conditions where mealtime, physical activity, sleep, and other living conditions are not constant daily. Other indicators may also detect slow baseline variations that are different from glucose spikes, for example, depending on time or activity.

Unexpectedly, multivariate analysis revealed a trend towards a positive correlation of medial plantar CNAP amplitude with HbA1c, suggesting that lower HbA1c may reflect a higher incidence of hypoglycemia, a potential risk factor for DPN. Araki et al. have reported that the incidence rate of hypoglycemia increased as HbA1c decreased in 1173 patients with type 2 diabetes [29, 30]. In our study, patients with hypoglycemia for more than 15 min had a tendency toward lower HbA1c levels. Furthermore, studies in rat models of diabetes have demonstrated that hypoglycemia can induce neuropathies [31–33].

DPN is a diabetes complication with an early onset. Postprandial elevated glucose, leading to increased glycemic variability, appears even in the early stage of diabetes and may explain the early onset of DPN. Increased glycemic variability has been shown to induce oxidative stress, leading to diabetes complications. Intermittent high glucose enhances apoptosis related to oxidative stress in human umbilical vein endothelial cells [34]. Oxidative stress, estimated from 24-h urinary excretion rates of free 8-iso prostaglandin F2alpha, was shown to be associated with MAGE obtained from CGM [35]. These findings suggest that increased glycemic variability might be associated with vascular damage via elevated oxidative stress.

This study had some limitations. First, the data obtained were cross-sectional. Although our analysis suggests an association of glycemic variability with DPN, it remains unclear whether amplified glycemic variability induces the development of DPN. Second, evaluation of glycemic variability may be insufficient based on only 7 days of CGM data. Thus, a future long-term longitudinal study is required. Finally, our study had a small sample size, and we were unable to evaluate patients with severe diabetes complications, which may have limited our ability to find significant associations of MAGE with NCS parameters other than the medial plantar nerve. An additional study in more patients with diabetes complications of varying severity is required.

## Conclusion

Glycemic variability in outpatients with diabetes was assessed using CGM, and DPN was quantitatively evaluated by NCS. We identified glycemic variability as an independent risk factor for DPN.

## Abbreviations

CGM: continuous glucose monitoring
DPN: diabetes peripheral neuropathy
DR: diabetes retinopathy
NCS: nerve conduction study
SNAP: sensory nerve action potential
CNAP: compound nerve action potential
CMAP: compound muscle action potential
SD: standard deviation
MAGE: mean amplitude of glycemic excursions
